# Moving psychiatric deinstitutionalization forward: A scoping review of barriers and facilitators

**DOI:** 10.1017/gmh.2023.18

**Published:** 2023-05-04

**Authors:** Cristian Montenegro, Matías Irarrázaval, Josefa González, Felicity Thomas, Jorge Urrutia

**Affiliations:** 1 Wellcome Centre for Cultures and Environments of Health, University of Exeter, Exeter, UK; 2 Millennium Institute for Research in Depression and Personality, Santiago, Chile; 3 Nursing School, Pontificia Universidad Católica de Chile, Santiago, Chile; 4 Section of Child and Adolescent Psychiatry, Pontificia Universidad Católica de Chile, Santiago, Chile; 5 Department of Psychiatry and Mental Health, Universidad de Chile, Santiago, Chile; 6 Departamento de Psicología, Universidad de Chile, Santiago, Chile

**Keywords:** Community-based initiatives, health care reform, severe mental illness, health policy, healthcare workers

## Abstract

Psychiatric deinstitutionalization (PDI) processes aim to transform long-term psychiatric care by closing or reducing psychiatric hospitals, reallocating beds, and establishing comprehensive community-based services for individuals with severe and persistent mental health difficulties. This scoping review explores the extensive literature on PDI, spanning decades, regions, socio-political contexts, and disciplines, to identify barriers and facilitators of PDI implementation, providing researchers and policymakers with a categorization of these factors. To identify barriers and facilitators, three electronic databases (Medline, CINAHL, and Sociological Abstracts) were searched, yielding 2,250 references. After screening and reviewing, 52 studies were included in the final analysis. Thematic synthesis was utilized to categorize the identified factors, responding to the review question. The analysis revealed that barriers to PDI include inadequate planning, funding, and leadership, limited knowledge, competing interests, insufficient community-based alternatives, and resistance from the workforce, community, and family/caregivers. In contrast, facilitators encompass careful planning, financing and coordination, available research and evidence, strong and sustained advocacy, comprehensive community services, and a well-trained workforce engaged in the process. Exogenous factors, such as conflict and humanitarian disasters, can also play a role in PDI processes. Implementing PDI requires a multifaceted strategy, strong leadership, diverse stakeholder participation, and long-term political and financial support. Understanding local needs and forces is crucial, and studying PDI necessitates methodological flexibility and sensitivity to contextual variation. At the same time, based on the development of the review itself, we identify four limitations in the literature, concerning “time,” “location,” “focus,” and “voice.” We call for a renewed research and advocacy agenda around this neglected aspect of contemporary global mental health policy is needed.

## Impact statement

The transition from a mental health system centered on long-term psychiatric hospital care to one centered on community-based services is complex, usually prolonged and requires adequate planning, sustained support and careful intersectoral coordination. The literature documenting and discussing psychiatric Deinstitutionalization (PDI) processes is vast, running across different time periods, regions, socio-political circumstances, and disciplines, and involving diverse models of institutionalization and community-based care. This scoping review maps this literature, identifying barriers and facilitators for PDI processes, developing a categorization that can help researchers and policymakers approach the various sources of complexity involved in this policy process. Based on the review, we propose five key areas of consideration for policymakers involved in PDI efforts: (i) needs assessment, design and scaling up; (ii) financing the transition; (iii) workforce attitudes and development; (iv) PDI implementation and (v) monitoring and quality assurance. We call for a multifaceted transition strategy that includes clear and strong leadership, participation from diverse stakeholders and long-term political and financial commitment. Countries going through the transition and those who are starting the process need a detailed understanding of their specific needs and contextual features at the legal, institutional, and political levels.

## Introduction

Starting during and after World War II in Western Europe and North America, psychiatric deinstitutionalization (PDI) is widely considered a central element of the modernization of psychiatry. It involves two broad components: (i) the closure or reduction of large psychiatric hospitals and (ii) the development of comprehensive community-based mental health services aiming to promote social inclusion and full citizenship for people living with severe mental illness A broad international consensus supports the need for a shift in mental health care, away from long-term institutionalization and toward comprehensive and integrated community-based and community-shaped services (Campbell and Burgess, 2012; WHO, 2013, 2021a; Thornicroft et al., 2016).

Significant economic, social, and cultural forces have precipitated the development of PDI, including public awareness of the dehumanizing effects of prolonged institutionalization in often poor conditions, the high cost of maintaining large, long-stay institutions, and pharmaceutical developments such as the introduction of psychotropic medication (Turner, 2004; Yohanna, 2013; Taylor Salisbury et al., 2016). For several decades, advocacy movements across the mental health and disability fields have demanded the protection of patients’ human rights, including the right to live independently in the community (Hillman, 2005; Mezzina et al., 2019). The UK, Italy, and Finland among other countries are generally regarded as good examples of PDI (Turner, 2004; Westman et al., 2012; Barbui et al., 2018). In the global south, while varying in approach and scale, Brazil, Chile, Sri Lanka and Vietnam have received praise for their efforts to move away from centralized psychiatric institutions (PAHO, 2008; Cohen and Minas, 2017).

Despite the consensus and the declarations by many governments, PDI remains a complex and fragile endeavor. Progress toward PDI varies greatly across and within countries (Hudson, 2019). In some regions, the majority of resources are still invested on centralized, long-term hospitalization (WHO and the Gulbenkian GMHP, 2014); in others, PDI has been delayed with the balance of mental health care shifting in favor of hospital-focused care (Sade et al., 2021); and in other cases, poor management of the PDI process has resulted in tragedy (see e.g., Moseneke’s, 2018 account of the Esidimeni tragedy in South Africa).

Understanding the factors that lead to or prevent the transition is crucial to inform the planning and implementation of PDI. Whilst these factors have been documented through the accounts of leaders and experts with hands-on experience, such as in the WHO’s Innovation in Deinstitutionalisation report (WHO and the Gulbenkian GMHP, 2014), there has been no previous attempt to systematically scope the literature on barriers and facilitators to PDI.

This paper therefore reports the results of a Scoping Review examining the extent and range of available research regarding barriers and facilitators involved in PDI processes. We organized the specific barriers in seven groups, and the facilitators in six groups, totaling 13 thematic groups. This categorization can be adapted to national realities and different levels of policy action around PDI, to guide research and policy efforts. The synthesis of this information allows us to establish a list of suggestions on ways to move forward.

## Methods

Given that the literature on this topic has not been comprehensively reviewed, the Scoping Review (ScR) (Arksey and O’Malley, 2005) methodology was used. The goal of a ScR is “to map rapidly the key concepts underpinning a research area and the main sources and types of evidence available (…), especially where an area is complex or has not been reviewed comprehensively before” (Mays et al., 2001, p. 194). For this review, a barrier to PDI was defined as any factor limiting or restricting the transition of care from long-term hospitalization to community-based services and supports. This may include, but is not limited to, issues related to the public-health priority agenda (Shen and Snowden, 2014); challenges in the implementation of mental health services in community settings (Kormann and Petronko, 2004; Saraceno et al., 2007); the resistance of workers employed by psychiatric institutions (Fakhoury and Priebe, 2002); and public and community responses, including stigma, paternalism and other sociocultural factors (Fisher et al., 2005; O’Doherty et al., 2016).

Correspondingly, we define a facilitator as any factor that fosters, promotes, or enables an adequate PDI process. These include the presence of well-organized social activism supporting the rights of persons with mental health problems (Anderson et al., 1998), the acceptance of mental illness as a human condition (Gostin, 2008), service paradigms that enhance social inclusion and citizenship (Fakhoury and Priebe, 2002; Saraceno, 2003) and political willingness (Saraceno et al., 2007).

This ScR was conducted following the Checklist for Preferred Reporting Items for Systematic reviews and Meta-Analyses extension for Scoping Review (PRISMA-ScR) (Tricco et al., 2018). A review protocol was created and registered at the Open Science Platform (doi: 10.17605/OSF.IO/XEBQW). See the protocol and PRISMA-ScR Checklist in Supplementary Materials A and B, respectively.

Three electronic databases were searched in May 2020 – Medline, CINAHL and Sociological Abstracts. Previously published systematic reviews on adults with severe mental health impairment (Lean et al., 2019; Richardson et al., 2019), barriers and facilitators to healthcare access (Adauy et al., 2013) and the deinstitutionalization process (May et al., 2019) informed our search strategy. The strategy combined terms across three dimensions: (i) adults with mental health impairment; (ii) barriers and facilitators related to health care delivery; and (iii) the deinstitutionalization process. The search strategy was not limited by study design or country. Tailored searches were developed for each database (see Supplementary Material C). Eligibility criteria were limited by studies in English and Spanish. All references obtained through the electronic database search and hand search were pooled in EndNote 11 (reference manager) and then uploaded to Covidence (screening and data extraction tool).

Studies selected for inclusion met the criteria detailed in Table 1. Initial eligibility was independently assessed by JU and JG based on title and abstract. At the level of full-text screening, a random sampling of 10% of the selected studies was pilot-tested (with three reviewers) to ensure at least 80% of agreement. Differences in opinions were discussed, and a final decision on their eligibility was made after discussion with CM. A specific data extraction form was created to record full study details and guide decisions about the relevance of individual studies (Table 2). Two reviewers (J.U.O. and J.G.M.) extracted data and checked for accuracy with another reviewer (C.M.C.). Eligibility criteria were further specified to differentiate and exclude specialized substance abuse services involving the legal system. Studies on child institutionalization and substance abuse were also excluded because of the distinct set of causes and challenges associated with these phenomena. Articles related to transinstitutionalization, the transfer of users from psychiatric hospitals to other institutional settings were excluded unless they addressed PDI barriers and facilitators directly.

**Table 1.** Inclusion and exclusion criteria

	Included	Excluded
Population	– Studies focused on adult users of long-term mental health services (stays longer than 60 days)	– Studies meeting the above criteria but where participants had a background of a long-term stay in Children Services facilities (children ward, orphans’ asylum, group home or residency) or specialized substance abuse services
Concept	Studies focused on providers, caregivers (family/friends) and users’ account on barriers and facilitators of the psychiatric deinstitutionalization process. Studies focused on PDI processes were included regardless of the study aims. Studies focused on reporting outcome measures related with the community mental health system where only included if they involved a reform process in the context of PDI	– No mention of any facilitator or barrier related to the process of PDI – Studies where the researchers could infer the presence of a barrier o facilitator of PDI but no direct link with PDI processes were clearly set out by the authors were excluded
Context	– Studies conducted in mental health setting – No restrictions were placed on the location of intervention delivery (i.e., hospital, day services, community health center, homes)	– No description of the mental health services provided
Type of Source	Published and unpublished (gray literature) sources including primary studies, textual papers, technical and governmental reports, calls to action, theoretical and political discussions, historical studies, book chapters and reviews	
Language	– Studies wrote in English or Spanish	– All other languages

*Note:* In the light of the potential differences that may affect the process of deinstitutionalization of Mental Health organizations from Social Services and Specialized Substance Abuse Services (like penal law involvement), this kind of interventions will be excluded.

**Table 2.** Data extraction form

Study Information	Correspondence Author
	Title
	Year of Publication
	Country in which the study was conducted
	Aim of study
	Study Design
	Population description
	Nº of participants
	Setting
	Provider type
Outcomes	Barriers to Psychiatric Deinstitutionalization
	Facilitators to Psychiatric Deinstitutionalization

During the research process, inclusion criteria adopted a dimensional character, with studies clearly stating barriers and facilitators on one extreme and studies where they had to be inferred, on the other. Given that ScR methodology is defined as an exploratory strategy to map the state of research on a topic (Arksey and O’Malley, 2005; Peters et al., 2015), no attempts were made to assess the methodological quality of the included studies.

Thematic synthesis (Thomas et al., 2004; Lucas et al., 2007; Thomas and Harden, 2008; Harden, 2010) of the selected papers followed a three-stage process. Firstly, it involved free coding the content of the text, to identify barriers and facilitators. Secondly, grouping and organizing the codes into an inductively developed set of categories. Finally, CM examined the categories and their respective codes in the light of the review question to produce an initial set of categories. The match between codes (barriers/facilitators) and categories, and their relevance for the review question was further discussed and refined through rounds of collective revision. A table with examples of the data coding process is available (Supplementary Material D).

To consistently scope the academic production around PDI over several decades, this review includes publications up until May 2020, intentionally excluding the literature related to the Covid-19 pandemic. To properly assess the effects of the Covid-19 pandemic upon processes of Deinstitutionalisation – and on the reality of long-term psychiatric hospitals in general – a different research question, and a tailored design is required.

## Results

**Figure 1.**
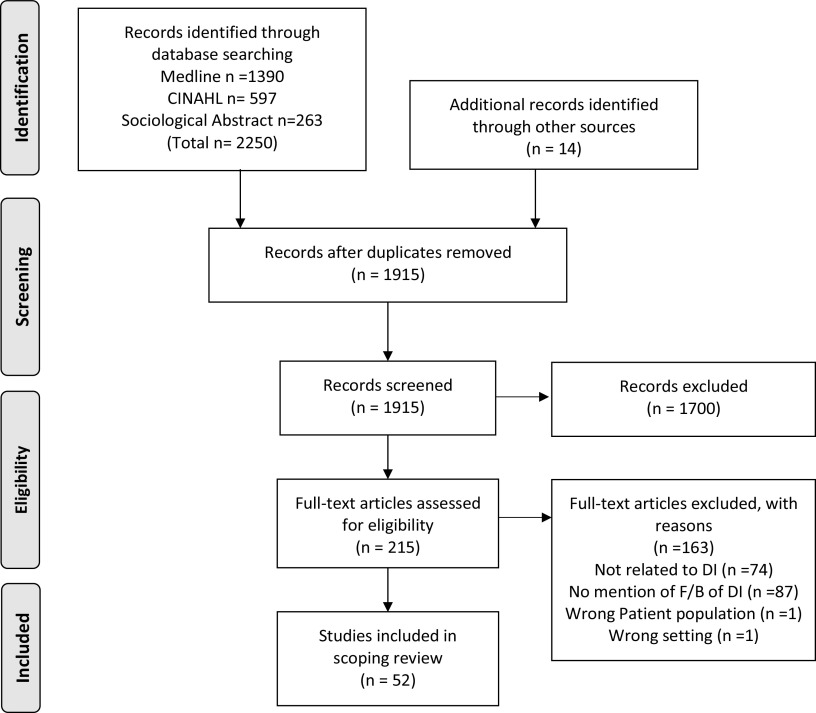
PRISMA 2009 flow diagram.

The search strategy retrieved 2,250 references. Nine more references were added after hand-searching reference lists and contacting relevant authors. After duplicate removal, 1,915 references were screened by title and abstract, leaving 215 articles for full-text screening. Finally, 52 studies were included in the analysis. Search results and the reasons for excluding full-text articles are provided in the PRISMA flowchart (Figure 1).

### Characteristics of the studies

**Figure 2.**
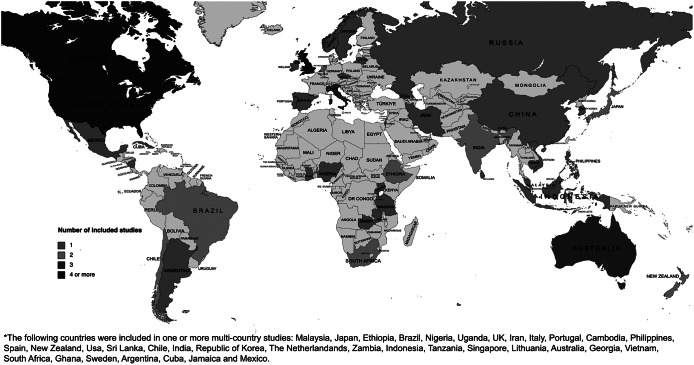
Geographical distribution of included studies. *Note:* The following countries were included in one or more multi-country studies: Malaysia, Japan, Ethiopia, Brazil, Nigeria, Uganda, UK, Iran, Italy, Portugal, Cambodia, Philippines, Spain, New Zealand, USA, Sri Lanka, Chile, India, Republic of Korea, The Netherlands, Zambia, Indonesia, Tanzania, Singapore, Lithuania, Australia, Georgia, Vietnam, South Africa, Ghana, Sweden, Argentina, Cuba, Jamaica and Mexico.

Included studies were published between 1977 and 2019. This broad temporal scope responds to the fact that an important proportion of research was parallel to the implementation of PDI policies in Europe and the USA during the 1970s and 1980s. Studies were predominantly conducted in the USA (*n* = 22), followed by the UK (*n* = 7) and Canada (*n* = 5). Figure 2 shows an overview of the geographical distribution of the included studies. Regarding the methodology, 25 publications were qualitative studies, 22 were quantitative, and 5 used mixed methods. We provide a summary of the studies’ characteristics in Table 3 and descriptions of each study in Table 4.

**Table 3.** Summary characteristics of included studies

		Nº of studies
Setting	Community mental health	19
	Mixed	15
	Inpatient	10
	Residency	4
	Primary care center	2
	Day Service	1
	Emergency Department	1
Provider	Public	30
	Other	16
	Private	5
	NGO	1
Language	English	52
	Spanish	0

**Table 4.** Study characteristics of included studies

References	Country	Aim of study	Setting	Study design	Type of end-user or participant	Number of participants	Provider type
Abas et al., 2003	New Zealand	To describe reasons for admission and alternatives to admission in a government-funded acute inpatient unit	Inpatient Unit	Mixed Methods	Adult patients admitted to a psychiatric hospitalization in the South Auckland Health Mental Health Services	255 admissions to an acute psychiatric unit in Auckland	Public
Aggett and Goldberg, 2005	UK	To describe the work of a busy Community Mental Health Team with outreach clients. Barriers to collaborative work and some of the team’s strategies to overcome them are delineated.	CMHC	Case series	Difficult to engage adult clients between 35 and 52 years old of an outreach community mental health team in an East London borough.	4 service users	Public
Alakeson, 2010	USA	To examine a range of innovative self-directed care programs in England, Germany, the Netherlands, and the United States.	CMHC	Narrative style	Home and community-based long-term care service users with physical and cognitive disabilities	Inapplicable	Public
Allen et al., 2007	UK	To investigate predictors for out of area placements for people with challenging behaviors and also reports on their costs and basic characteristics.	Mixed	Descriptive Transversal	All people attending to services supporting children and adults with intellectual disability in a large area of South Wales in conjunction with health, education, unitary authority, voluntary and private sector commissioners and providers	1,458 people	Public
Anderson et al., 1998	USA	To show the changes over 30 years in state institutional populations, interstate variability, movement of individuals into and out of state institutions, costs of state institutional care, and state institution closure as a result of social policy	Inpatient Unit	Descriptive Longitudinal	Patients in institutions for mental disabilities and epileptics between 1950 and 1968	Inapplicable	Public
Ash et al., 2015	Australia	To describe the implementation of recovery-based practice into a psychiatric intensive care unit, and report change in seclusion rates over the period when these changes were introduced (2011–2013)	Inpatient Unit	Mixed Methods	Consumers (average age 38 years) detained under the SA Mental Health Act. Eleven percent had been charged with or convicted of an offense with a custodial sentence. Common diagnoses were schizophrenia (32%), drug-induced psychosis (18%) and bipolar disorder (manic) (18%). The average length of stay was 11.5 days	63 people	Public
Barry et al., 2002	USA	To examine the relationship between age, the use of health services and level of functioning in patients with schizophrenia across the adult lifespan	Mixed	Descriptive Transversal	Veterans with schizophrenia drawn from the VA National Psychosis Registry who received a diagnosis of schizophrenia during a VA clinical encounter between 1999 and 2000	102.256	Other: not described
Barton, 1983	USA	To discuss the role of mental hospital in the health care system for the elderly	Inpatient Unit	Narrative style	Inapplicable	Inapplicable	Mixed
Bennett, 1983	UK	To describe factors that fostered the deinstitutionalization process in the UK and its consequences in psychiatric services	Mixed	Narrative style	Inapplicable	Inapplicable	Public
Bredenberg, 1983	USA	To present available documentation regarding the implications of residential integration of geriatric ex-mental patients and the well elderly and make recommendations for future action	Residency	Narrative style	elderly discharged mental health service users	Inapplicable	Other: not described
Bryant et al., 2004	UK	To identify how the experience of attending day services met the needs of people with enduring mental health problems	Day Unit	Thematic analysis	patient population	39 people	Public
Chakraborty et al., 2011	UK	To compare measures of perceived racism, medication adherence and hospital admission between African- Caribbean and white British patients with psychosis	Mixed	Cohort study	participants aged 18–65 years; with a self-assigned ethnicity of Caribbean origin with either parents or grandparents born in the Caribbean; having a Research Diagnostic Criteria-defined psychotic symptom and in receipt of psychiatric services in north London, UK	110 people	Public
Chan and Mak, 2014	Hong Kong	To examine the mediating role of self-stigma and unmet needs in the relationship between psychiatric symptom severity and subjective quality of life	CMHC	Case series	Adults with schizophrenia spectrum disorders attending community mental health services in Hong Kong	400	Nonprofit organization
Chopra and Herrman, 2011	Australia	To assess the long-term outcomes for the original cohort of 18 residents of the Footbridge Community Care Unit (CCU), a residential psychiatric rehabilitation unit at St Vincent’s Mental Health Melbourne	ED	Cohort study	14 schizophrenic and 4 people with schizoaffective disorder	18	Public
Cohen, 1983	USA	To clarify conceptions about mental illness in later life and promote the development of mental health services for the elderly in the community	Residency	Narrative style	senior people with mental health difficulties living in housing arrangements	Inapplicable	Other: not described
Conway et al., 1994	UK	To report outcomes of community mental health services for people with schizophrenia who had shown very low levels of supported housing and structured day activity	CMHC	Cohort study	patients from West Lambeth, London originally aged 20–65 years who satisfied the research diagnostic criteria for schizophrenia	51 people	Other: not described
Dorwart et al., 1991	USA	To assess the effect of changes in ownership and types of inpatient settings on the structure of the mental health services system	Inpatient Unit	Analytic transversal	All nonfederal psychiatric hospitals in the United States, including community mental health centers with inpatient units between October 1987 and May 1988	915 hospitals	Mixed
Evans et al., 2012	USA	To describe the conversion of partial hospitals into recovery-oriented programs as part of system transformation	CMHC	Narrative style	Stakeholders involved in a transformation of mental health service in a hospital	Inapplicable	Other: not described
Fakhoury and Priebe, 2002	UK	To provide an international overview of deinstitutionalization and review related issues as discussed in the current literature	Mixed	Narrative style	Inapplicable	Inapplicable	Mixed
Freedman and Moran, 1984	USA	To identify and discuss the major policy issues related to the care of the chronically mentally ill, specifically the effects and implications of deinstitutionalization for this particular population	CMHC	Case report	A 32-year-old schizophrenic who has spent more than 10 years in mental health institutions	Inapplicable	Public
Goering et al., 1984	Canada	To describe the 6-month and 2-year postdischarge outcome in each of five aftercare components for 505 subjects in a traditional system of service delivery	Inpatient Unit	Cohort study	Adult people discharged from inpatient units in Toronto	505 participants	Public
Grabowski et al., 2009	USA	To estimate the cross-state variation in the proportion of nursing home admissions indicating a mental illness, and the proportion of persons with mental illness admitted to nursing homes	Residency	Descriptive Transversal	Nursing home admissions in the USA during 2005	1.150.734 new admissions	Private
Huang et al., 2017	Singapore	To design a general practitioner– partnership programme in an institute in Singapore to facilitate the transition to community services and gauge the impact of the interventions chosen to improve uptake of referrals	CMHC	Mixed Methods	Stable mental health service users referred to the GP from December 2014 to January 2016partnership programme in a mental health institute in Singapore	238 service users	Private
John et al., 2010	India	To describe the successful management of a person with schizophrenia in the community through a primary care team in liaison with psychiatrist services	CMHC	Case report	adult with psychotic symptoms living in an urban area of India	1 person	Public
Kaffman et al., 1996	Israel	To report on an alternative community care program that has been developed and implemented in the Kibbutz Clinic for the treatment and rehabilitation of the severely mentally ill	CMHC	Mixed Methods	adult people with a severe mental illness with poor functioning who participated in the program conducted in Telem, Israel, for at least 18 months and followed up for a minimum of 4 years	124 patients	Private
Kalisova et al., 2018	Czech Republic	To assess the effect of the S.U.P.R. psychosocial rehabilitation programme on the quality of care at the longer-term inpatient psychiatric departments	Inpatient Unit	Experimental not randomized (“before and after” design)	All Czech psychiatric hospitals focused on longer-term inpatients, mainly with a diagnosis of schizophrenia	14 units for 499 patients with severe mental illness with complex needs	Other: not described
Yip, 2006	China	To review and evaluate the implementation of community mental health in the People’s Republic of China	CMHC	Narrative style	Inapplicable	Inapplicable	Public
Kleiner and Drews, 1992	USA and Norway	To describe the experiences in the creation of innovative service delivery system which integrates psychiatric services with lay community support systems and patient social networks	CMHC	Narrative style	Psychotic patients who had more than two years of cumulative hospitalization, and who could not be placed with relatives	Inapplicable	Other: not described
Kraudy et al., 1987	Nicaragua	To assess the extent to which the new proposed model had been translated into a different way of delivering psychiatric care in Nicaragua	Primary Care Centre	Descriptive Transversal	children and adult patients attending one of the surveyed services for the first time irrespective of whether or not they had a psychiatric history	342 patients	Public
Lamb and Goertzel, 1977	USA	To assess the career of psychiatrically disabled people in the community	CMHC	Descriptive Transversal	Long-term psychiatrically disable people between 18 and 64 years old who live in the community in California with a psychotic diagnoses	99 people	Private
Lavoie-Tremblay et al., 2012	Canada	To describe how families and decision-makers perceive collaboration in the context of a major transformation of mental health services and to identify the factors that facilitate and hinder family collaboration	CMHC	Thematic analysis	family members of users of mental health services and key decision makers on the mental health service	54 family members and 22 decision-makers	Public
Mallik et al., 1998	USA	To identify perceived barriers to community integration in people with psychiatric disabilities, in the areas of skills, environmental support, and community resources	Inpatient Unit	Case series	People with psychiatric disabilities in the Alliance of Psychiatric Rehabilitation Program in Baltimore County, Maryland	42 people	Public
Manuel et al., 2012	USA	To explore the experience of women with severe mental illness in transition from psychiatric hospital care to the community	Residency	Thematic analysis	women living in transitional residences on the grounds of two state-operated psychiatric hospitals in the New York City metropolitan area, awaiting discharge to both supervised and independent housing in New York City	25 women	Public
Matsea et al., 2019	South Africa	To explore the views of different stakeholders about their roles as support systems for people with mental illness and their families in a rural setting	CMHC	Content Analysis	Stakeholders comprising traditional health practitioners (faith and traditional healer), traditional leaders, church members, home-based care team and police officers from Mashashane, a rural setting in Limpopo Province, South Africa	41 stakeholders	Public
Mayston et al., 2016	Ethiopia	To engage key stakeholders in participatory planning for a shift to mental health care integrated into primary care, and to explore their perspectives on acceptability and feasibility of the change	CMHC	Framework analysis	key stakeholders (healthcare administrators and providers, caregivers, service users and community leaders) living in Butajira town	11 service users, 27 caregivers, 15 health extension worker and 10 health center workers	Public
McCubbin, 1994	Canada	To reevaluate the recent tendency to attribute economic causes to deinstitutionalization and its subsequent “treatment in the community” mental health systems	Mixed	Narrative style	Inapplicable	Inapplicable	Mixed
Mechanic and Rochefort, 1990	USA	To provide a comprehensive overview of the causes, nature, and consequences of the practice of deinstitutionalization in the United States	Mixed	Narrative style	Inapplicable	Inapplicable	Mixed
O’Doherty et al., 2016	Ireland	To document the views of family members of people with an intellectual disability regarding implementation of a personalized model of social support in Ireland	CMHC	Grounded theory	parent, adult sibling or extended family member of a person receiving full-time residential supports from the agency	40 family members	Public
Oshima and Kuno, 2006	Japan	To explore how the introduction of community-based care has changed the role of psychiatric hospitals and families in caring for people with mental illness by examining the different types of living settings of clients treated for schizophrenia in Kawasaki as compared with a similar group of clients nationally	CMHC	Descriptive Transversal	adults with a diagnosis of schizophrenia living in the community and hospitalized in Kawasaki and the rest of Japan	3.845 people living in Kawasaki and 448.000 living in Japan	Private
Paho, 2008	Argentina, Brazil, Chile, Cuba, Jamaica and Mexico	To convey some of the more innovative experiences to reform mental health services implemented in Latin America and the Caribbean	Mixed	Narrative style	Inapplicable	Inapplicable	Public
Rizzardo et al., 1986	Italy	To analyze the impact of the reform on health care delivery by the general practitioner in an urban district in the Veneto region	Primary Care Centre	Descriptive Transversal	General practitioners working in a psychiatric service run by the University of Padua by 1983	24 general practitioners	Other: University facilities
Rose, 1979	USA	To analyze deinstitutionalization policy on the sector of community mental health care and review its accomplishments and difficulties	Mixed	Narrative style	Inapplicable	Inapplicable	Mixed
Rosenheck, 2000	USA	To review the relationship between mental health service delivery and the community in which it is embedded	CMHC	Narrative style	Inapplicable	Inapplicable	Mixed
Schmidt, 2000	Canada	To examine how psychiatric rehabilitation fits within a remote First Nations community	CMHC	Thematic analysis	service providers, consumers and family members of aboriginal people with severe mental illness living in northern British Columbia	10 stakeholders	Public
Semke, 1999	USA	To explore system outcomes of interventions that were aimed at lowering high use of long-stay state hospitals	Mixed	Descriptive Transversal	adults living in the Washington state who experienced one psychiatric hospitalization of 30 days or more, or three or more psychiatric hospital admissions during a “pre-reform” period (1988) or after implementation of reform interventions (between 1991 and 1993)	2.646.307 high utilizers of state hospitals	Public
Shen and Snowden, 2014	USA	To examine whether the institutionalization of deinstitutionalization policy changed the supply of psychiatric beds in 193 countries from 2001 to 2011	Inpatient Units	Ecological study	Mental health systems as units	193 countries	Public
Stelovich, 1979	USA	To describe factors related to deinstitutionalization leading to transfer mental health service delivery from civil mental health hospitals to prison facilities	Inpatient Unit	Narrative style	Psychiatric patients transferred to prison facilities in Massachusetts	Inapplicable	Public
Swidler and Tauriello, 1995	USA	To describe the political processes leading to the Community Mental Health Reinvestment Act passage, the obstacle overcome by legislative negotiators and implementation issues	Mixed	Narrative style	Inapplicable	Inapplicable	Public
Sytema et al., 1996	Italy and Netherlands	To compare the treatment of severely mentally ill patients in a community mental health service without the back-up of a mental hospital with the treatment provided in an institution-based system in which mental hospital are still predominant	Mixed	Cohort study	Patient with schizophrenia that contacted a service at least once in 1988 or in 1989 in Groningen (The Netherlands) or South Verona (Italy)	812 patients	Mixed
Wasylenki and Goering, 1995	Canada	To describe the authors’ involvement in three service delivery projects in Ontario and discuss how, by assuming multiple roles, they were able to ensure that planning and policy development were informed by current knowledge	Mixed	Narrative style	Inapplicable	Inapplicable	Public
Weiss, 1990	USA	To analyze deinstitutionalization policies implemented in 1946 and 1963 in USA	Mixed	Narrative style	Inapplicable	Inapplicable	Public
WHO, 2014	Malaysia, Japan, Ethiopia, Brazil, Nigeria, Uganda, UK, Iran, Italy, Portugal, Cambodia, Philippines, Spain, New Zealand, USA, Sri Lanka, Chile, India, Republic of Korea, The Netherlands, Zambia, Indonesia, Tanzania, Singapore, Lithuania, Australia, Georgia, Vietnam, South Africa, Ghana, Sweden	To capture lessons learnt from those who have been involved directly with deinstitutionalization and/or expanding community-based services and identify innovative strategies and methods associated with success of this process	Mixed	Mixed Methods	mental health experts involved directly with deinstitutionalization and/or expanding community-based services	78 people	Public

Abbreviations: CMHC, Community Mental Health Centre; ED, Emergency Department.

It is important to consider that this is a general categorization based on the available literature, whose aim is to identify what has been reported as a barrier and as a facilitator in a systematically selected, diverse set of references. We applied thematic analysis to the entire set, and on that basis, we developed this initial categorization. We are not establishing the prevalence of each barrier/facilitator across the set or contrasting the characteristics of each barrier/facilitator across regions or within a specific stage in the PDI process. For specific information about the composition of the categories and codes, see Table 5 for barriers and Table 6 for facilitators.

**Table 5.** Barriers to the process of psychiatric deinstitutionalization

Category	Descriptive themes	References
1. Planning, leadership, and funding	Mental health policy: Responsibility/accountability	Rose, 1979
	Reform fragility: charismatic leadership	PAHO, 2008
	Reform fragility: Lack of synchronization between bed reduction and development of CBMHSs	Freedman and Moran, 1984; Shen and Snowden, 2014
	Reform fragility: Unaccountability of failure	Rose, 1979; Freedman and Moran, 1984; Rosenheck, 2000
	Funding: Continuity of community care	Mechanic and Rochefort, 1990; McCubbin, 1994; PAHO, 2008
	Funding: Hospital funds not reallocated to CMHS	Fakhoury and Priebe, 2002; PAHO, 2008
2. Knowledge/Science	Conceptual limitations and ambiguities	Bennett and Morris, 1983; Freedman and Moran, 1984; McCubbin, 1994; Mallik et al., 1998; Fakhoury and Priebe, 2002
	Evidence: Lack of evidence on DI processes	Shen and Snowden, 2014
	Lack of research and innovation on alternatives to institutionalization	Bennett and Morris, 1983
3. Power, interests, and influences	Irrelevance of Mental Health in the political/policy agenda	Mechanic and Rochefort, 1990; Semke, 1999; PAHO, 2008
	Market factors fostering reinstitutionalization	Rose, 1979; Barton, 1983; Freedman and Moran, 1984; Dorwart et al., 1991; Fakhoury and Priebe, 2002
	Uncoordinated and fragmentary advocacy actions.	Mechanic and Rochefort, 1990; McCubbin, 1994; Rosenheck, 2000
	Vested interests: Pharmaceutical	McCubbin, 1994
4. Services and supports in the community	Centralized System	Kleiner and Drews, 1992
	Patients: Challenging behaviors	Allen et al., 2007
	Patients: Old Age	Barry et al., 2002
	Services: Hospital-centric models and practices	Bennett and Morris, 1983; Kaffman et al., 1996
	Early discharge	Stelovich, 1979; Kleiner and Drews, 1992
	Services: Lack of services and support in the community	Weiss, 1990; McCubbin, 1994; Fakhoury and Priebe, 2002; Oshima and Kuno, 2006
	Housing: Inadequate, insufficient	Mechanic and Rochefort, 1990; PAHO, 2008; Grabowski et al., 2009
	Dependence on disability benefits and/or pensions	Freedman and Moran, 1984; Chopra and Herrman, 2011; Manuel et al., 2012
	Patients: Disempowerment/Fatalism	Chopra and Herrman, 2011
	Insufficient Public Support	Manuel et al., 2012
	Patients: No money	Goering et al., 1984
	Clashing views on DI within the Workforce	Kleiner and Drews, 1992; PAHO, 2008
5. Workforce	Shortages in general	Schmidt, 2000; Fakhoury and Priebe, 2002; Shen and Snowden, 2014; WHO, 2014
	Shortages of specific professions	Ash et al., 2015
	Inadequate training	Barton, 1983; PAHO, 2008; WHO, 2014; Mayston et al., 2016
	Moral concerns and fears	Kleiner and Drews, 1992; PAHO, 2008; Ash et al., 2015
	Pessimism	Cohen, 1983; Kleiner and Drews, 1992; Aggett and Goldberg, 2005
	Practices of exclusion	Bryant et al., 2004; Chakraborty et al., 2011
	Stigma in workforce	Barton, 1983; Semke, 1999
	Vested interests: Workforce	Swidler and Tauriello, 1995; Shen and Snowden, 2014
6. Communities and the public	Communities are hostile toward users	Bredenberg, 1983; Fakhoury and Priebe, 2002; Aggett and Goldberg, 2005; PAHO, 2008; O’Doherty et al., 2016
	Communities are ill-prepared to integrate users	Bredenberg, 1983; Fakhoury and Priebe, 2002
	Public acceptance of social control	Swidler and Tauriello, 1995; Fakhoury and Priebe, 2002; Allen et al., 2007
	Stigma and self-stigma	Mechanic and Rochefort, 1990; Fakhoury and Priebe, 2002; Aggett and Goldberg, 2005; ; PAHO, 2008Manuel et al., 2012; Chan and Mak, 2014; O’Doherty et al., 2016
7. Family/Carers	Broken ties between families and services	Aggett and Goldberg, 2005
	Lack of support and/or unfair expectations toward families	Barton, 1983; Mechanic and Rochefort, 1990; Oshima and Kuno, 2006; ; Yip, 2006; Lavoie-Tremblay et al., 2012
	Skepticism and Opposition from families	McCubbin, 1994; Oshima and Kuno, 2006

**Table 6.** Facilitators to the process of psychiatric deinstitutionalization

Category	Descriptive themes	References
Planning, Leadership and Funding/Economic aspects	Centralized governance of the process	PAHO, 2008
	Austerity and fiscal pressure	PAHO, 2008
	Disability insurance	Mechanic and Rochefort, 1990
	Economic incentives for DI	Mechanic and Rochefort, 1990
	Fiscal strain on state mental hospital	Mechanic and Rochefort, 1990; O’Doherty et al., 2016
	International policy networks and advocacy	PAHO, 2008
	Intersectoral alliances and coordination	PAHO, 2008
Knowledge/Science	Available evidence about alternatives	Weiss, 1990
	Conceptual Clarity	Freedman and Moran, 1984; Kleiner and Drews, 1992; McCubbin, 1994
	Documented Experience	Shen and Snowden, 2014
	Evidence of human rights violations	PAHO, 2008
	Intellectual cross-fertilization toward CBSs	Mechanic and Rochefort, 1990; PAHO, 2008
	Knowledge of effects of institutions on individual patients	Bennett and Morris, 1983; Mechanic and Rochefort, 1990; Kleiner and Drews, 1992; Anderson et al., 1998
	Psychopharmacological developments	Bennett and Morris, 1983; Bredenberg, 1983; Freedman and Moran, 1984; Mechanic and Rochefort, 1990; Weiss, 1990; Kleiner and Drews, 1992; Anderson et al., 1998
Power, interests and influences	Human rights legislation	Anderson et al., 1998; PAHO, 2008
	Influence of civil rights movements	Mechanic and Rochefort, 1990; PAHO, 2008
	Legal limitations to commitment/coercion	Freedman and Moran, 1984; Mechanic and Rochefort, 1990;
	Legal push toward community-based treatments	Freedman and Moran, 1984
	Legal standards for facility construction/operation	Anderson et al., 1998
	MH Legislation	Freedman and Moran, 1984; PAHO, 2008; Shen and Snowden, 2014
	Advocacy from professional organizations/groups	Weiss, 1990; WHO, 2014
	International policy pressure	Shen and Snowden, 2014
Services and supports in the community	Service-user movements and demands	Kleiner and Drews, 1992; Anderson et al., 1998
	Comprehensive and structured network of CB services	Lamb and Goertzel, 1977; Cohen, 1983; Conway et al., 1994; Evans et al., 2012
	Continuity of care	Sytema et al., 1996
	Income for patients	Alakeson, 2010
	Individualization of care in the community	Kalisova et al., 2018
	Integration of mental health in PHC	Kraudy et al., 1987; PAHO, 2008; Evans et al., 2012; John et al., 2010
	Limit readmission by closing beds	PAHO, 2008
	Recovery-based services in a psych ICUs	Ash et al., 2015
	Scale up of outpatient services	Bennett and Morris, 1983; Abas et al., 2003
	Self-directed support: Autonomy in the use/selection of services	Alakeson, 2010
	Shared decision-making and service user involvement	Chan and Mak, 2014
	Supporting PHC expertise to raise service-user confidence	Huang et al., 2017
	Social Help	Lamb and Goertzel, 1977
Workforce	Anti-stigma practice	Mayston et al., 2016; Huang et al., 2017; Matsea et al., 2019
	PHC training	PAHO, 2008
	WF training	Weiss, 1990; Wasylenki and Goering, 1995
Exogenous factors	Exogenous shocks (disasters, war)	Stelovich, 1979
	Redemocratization	Rizzardo et al., 1986

### Barriers to the process of psychiatric deinstitutionalization

Barriers to the process were organized under seven categories, summarized in Table 5 and described in detail below.

#### Planning, leadership and funding

This category includes barriers related to design, implementation, monitoring and overall leadership of the process, and its interaction with other policy processes. One barrier is the lack of accountability from the government to carry out the reform properly, refusing responsibility for housing, social or medical needs and not including other agencies in patient discharge planning (Rose, 1979). The absence of clear operational goals may hinder performance evaluation (Rosenheck, 2000). Charismatic and ideologically driven leadership is important at the beginning, although is vulnerable to political shifts, including elections and changes in government (PAHO, 2008).

Barriers related to funding included the lack of a clear policy that assured the reallocation of resources from hospitals to CMHS (Fakhoury and Priebe, 2002; PAHO, 2008) and a lack of funding to ensure the continuity of community services (Mechanic and Rochefort, 1990; McCubbin, 1994; PAHO, 2008). This is to secure a synchronicity between downsizing psychiatric hospitals and the scaling up of psychosocial interventions.

#### Knowledge/science

Conceptual barriers to promoting PDI were identified. Some authors consider that the lack of research on PDI processes (Bennett and Morris, 1983), paralyze or slow down policy planning and implementation (Shen and Snowden, 2014). At the conceptual level, reducing the concept of community care to narrow geographical proximity can limit the development of community-based interventions (Bennett and Morris, 1983).

Some authors criticized the inadequate transfer and use of certain service paradigms, such as the application of urban-centered interventions to rural locations (Kraudy et al., 1987) without previous identification of rural specificities, creating a disconnection between users and facilities (Schmidt, 2000).

#### Power, interests and influences

Barriers related to the conflict between the interests and perspectives of different groups were grouped under this category.

Authors have discussed the impact of the privatization of mental health care in the wake of the closure of psychiatric hospitals. Market-driven decisions can recreate similar conditions to those in old psychiatric facilities (Rose, 1979). The rise of private hospitals in the United States and their reluctance to participate in non-profit services, such as working with existing public providers, influences access to and the nature of mental health care. Private for-profit hospitals may restrict access to care for uninsured patients (Dorwart et al., 1991). Additionally, private insurance in the United States often encourages unnecessary hospitalization and discourages psychosocial interventions and alternative forms of treatment (Barton, 1983; Freedman and Moran, 1984).

Furthermore, the low cost of hospitalization in some areas, as reported in Asia (Fakhoury and Priebe, 2002), does not provide an economic incentive to push for deinstitutionalization.

The dependence of psychiatric research and development on drug-companies is seen as a barrier. McCubbin stated that the vested interests of the pharmaceutical industry may influence psychiatric practice by selectively supporting medical schools, conferences, and journals, potentially tuning the vision of community mental health into a market opportunity (McCubbin, 1994).

Finally, the lack of relevance of mental health in the political agenda is a crucial, over-encompassing barrier to effective advocacy efforts (Mechanic and Rochefort, 1990; Semke, 1999; PAHO, 2008), as is the uncoordinated and fragmentary nature of these efforts (Mechanic and Rochefort, 1990; McCubbin, 1994; Rosenheck, 2000).

#### Services and support in the community

The slow development of community programmes forced patients to return to long-term institutions, risking chronification (Kaffman et al., 1996). There have been reports of problems caused by the sudden decrease in psychiatric beds without corresponding increases in community-based services. This can result in unintended transfers of patients to other institution-based services and even imprisonment (Shen and Snowden, 2014). Inadequate training of community-based workers, discharge without community support (Shen and Snowden, 2014) and early release promoted by legislatively mandated PDI policies (Kleiner and Drews, 1992) are elements to consider.

The authors identified several barriers to adequate integration of discharged users into their communities, including the absence of jobs and income (Goering et al., 1984), inadequate housing (Grabowski et al., 2009), and insufficient public support (Manuel et al., 2012). Other barriers included challenging behaviors (Allen et al., 2007), old age (Barry et al., 2002), and pessimistic attitudes and feelings of disempowerment and hopelessness among patients (Chopra and Herrman, 2011). In addition, the decrease in disability pensions following an increase in earned income was also identified as a barrier to social integration, as it can discourage work (Chopra and Herrman, 2011).

#### Workforce

Barriers related to the workforce in both institutionalized settings and community services were identified. Regarding human resources, authors mentioned staff shortages as a barrier for the transition toward community-based care (Rose, 1979; Stelovich, 1979; Fakhoury and Priebe, 2002; Shen and Snowden, 2014). Another barrier reported was the internal frictions and the existence of opposing views about care and rehabilitation (Kaffman et al., 1996; O’Doherty et al., 2016). More specifically, the psychiatric hospital workforce can delay or hinder the transformation of psychiatric institutions for fear of losing their livelihoods (Swidler and Tauriello, 1995; Shen and Snowden, 2014). Workers can express reluctance and skepticism regarding the feasibility of community living for institutionalized persons (Mayston et al., 2016; O’Doherty et al., 2016). This includes the development of unfair expectations toward family members, which alienated carers and hindered their willingness to accept responsibility (Barton, 1983).

On the other hand, service providers located in the community can be sources of stigma, expressed in the avoidance of formerly institutionalized patients (Barton, 1983), hopelessness toward treatment (Aggett and Goldberg, 2005), exclusion of users from constructing their treatment plan (Bryant et al., 2004) and fears stemming from the lack of restraining measures (Ash et al., 2015). Perceived racism at the hands of service providers can lead to mistrust in patients, causing them to either reject treatment or have poor adherence, which in turn can result in poorer outcomes, such as a longer hospital stays (Chakraborty et al., 2011).

#### Communities and the public

Factors limiting social inclusion, comprising attitudes toward persons with SMI and community responses to PDI processes, were grouped under this category. Lack of preparation and stigma (Bredenberg, 1983; Mechanic and Rochefort, 1990; Fakhoury and Priebe, 2002; Aggett and Goldberg, 2005; PAHO, 2008; Manuel et al., 2012; Chan and Mak, 2014; O’Doherty et al., 2016) leads to hostile attitudes toward service-users challenging social integration (Bredenberg, 1983; Fakhoury and Priebe, 2002; Aggett and Goldberg, 2005; PAHO, 2008; O’Doherty et al., 2016). The attribution of dangerousness to individuals with SMI and the public acceptance of social control measures over recovery-oriented alternatives were also reported as barriers to PDI processes (Fakhoury and Priebe, 2002; Matsea et al., 2019).

#### Family/carers

Authors highlighted the difficulties in maintaining relationships between caregivers and community services (Barton, 1983; McCubbin, 1994; Aggett and Goldberg, 2005; Yip, 2006; Lavoie-Tremblay et al., 2012; Mayston et al., 2016; O’Doherty et al., 2016). Previous experiences of failed treatments can lead to lack of cooperation and hostility toward services (Aggett and Goldberg, 2005). Professionals can be reluctant to cooperate and skeptical about the feasibility of community living (Mayston et al., 2016; O’Doherty et al., 2016). Families and caregivers may have concerns about community living and its suitability for people with high support needs (O’Doherty et al., 2016) and concerns about receiving the burden of care, and this can alienate them and hinder their willingness to accept responsibility.

### Facilitators to the process of psychiatric deinstitutionalization

Facilitators in the process were organized under six categories summarized in Table 6 and described in detail below.

#### Planning, leadership and funding

Factors related to organizational and managerial capacities required for the transition were grouped under this category. Authors stated that the presence of a central mental health authority increased the potential to ensure effective coordination. For example, Latin America and Caribbean countries have developed mental health units within the health ministry capable of overseeing coordination (PAHO, 2008). Coordination across countries in the initial phases of reform played a crucial role, by sharing technical support and experiences of implementation (PAHO, 2008). Authors highlighted the relevance of developing intersectoral coordination, which may act as a safety net for persons with serious mental health illness reducing acute episodes (PAHO, 2008).

Studies mentioned how increases in psychiatric population and fiscal strain on state mental hospitals drove governments to develop an alternative mental health strategy (Mechanic and Rochefort, 1990; McCubbin, 1994). The pressure on fiscal resources -partly linked to economic crisis- made the costs of mental health hospitals and their inefficiency more apparent (PAHO, 2008). Also, the direct transference of funds – from reduced hospital expenditure – to community-based services was mentioned as a factor that fostered the transference of patients from state hospitals to alternative placements in the community (Mechanic and Rochefort, 1990). Finally, the growth of disability insurance was understood as a facilitator of the process of discharging service users from psychiatric hospitals by contributing to their support in the community (Mechanic and Rochefort, 1990).

#### Knowledge/science

Interdisciplinary research focusing on the legal and economic factors which influence PDI processes and practices was valued (Mechanic and Rochefort, 1990; PAHO, 2008). The elucidation of adverse effects of institutions on individual patients (Bennett and Morris, 1983; Mechanic and Rochefort, 1990; Kleiner and Drews, 1992; Anderson et al., 1998) together with the documentation of human rights violations in mental health hospitals helped in catalyzing the reform process (Bennett and Morris, 1983; PAHO, 2008). More generally, some authors stressed that conceptual clarity regarding the application of a biopsychosocial model to the mental health field (McCubbin, 1994) and the interpersonal aspect of mental health (Bennett and Morris, 1983; Kleiner and Drews, 1992) helped in the rolling up of the Deinstitutionalisation processes.

In the early stages of PDI in the USA, the allocation of research grants to state mental health hospitals developing pilot testing of outpatient treatment and rehabilitation helped in the shift of funds from mental hospitals into general hospitals (Weiss, 1990). The dissemination of early experiences of innovative policy implementation in mental health facilitated the adoption of Deinstitutionalisation practices in other regions (Shen and Snowden, 2014). Finally, the development of psychotropic medication and the reduction of psychiatric symptomatology helped to build trust in the implementation of less coercive management plans that were feasible to apply at the community level (Bennett and Morris, 1983; Bredenberg, 1983; Freedman and Moran, 1984; Mechanic and Rochefort, 1990; Kleiner and Drews, 1992; Anderson et al., 1998).

#### Power, interests and influences

This category points to the role of social movements and organizations in influencing the development of Deinstitutionalisation processes. This includes advocacy actions and legal transformations.

Mental health professional groups and civil society organizations were seen as key agents contributing to overcome stigma and change the delivery of mental health services (Weiss, 1990). Some authors emphasized the importance of promoting the active involvement of civil society groups (Oshima and Kuno, 2006). Finally, authors highlight how the internationalization of mental health reforms puts increasing pressure on other countries to jump on the “bandwagon” to avoid appearing antiquated (Shen and Snowden, 2014).

Recognition of the rights of people with disabilities and their defense by civil rights movements fostered the development of new mental health laws promoting less restrictive therapeutic alternatives and broader transformations on mental health systems (Freedman and Moran, 1984; Mechanic and Rochefort, 1990; Anderson et al., 1998; PAHO, 2008; Shen and Snowden, 2014). These changes involved expanding the supply of options in the community (Freedman and Moran, 1984; Mechanic and Rochefort, 1990; Anderson et al., 1998; PAHO, 2008) and relocating investment from institutions to community services (Swidler and Tauriello, 1995). In some countries, an extensive and strong network of community-based organizations provided opportunities for community participation, facilitating the effective integration of patients into the community (PAHO, 2008). This was accompanied by the divulgation of reports showing mistreatment of patients in hospitals, pushing public sensitivity against asylums (Anderson et al., 1998).

#### Services and supports in the community

This category describes how the characteristics and distribution of community-based services and support for persons with SMI acted as facilitators in PDI processes.

Authors noted how policies around prevention in mental health, the integration of mental health services in primary health care centers (Kraudy et al., 1987; PAHO, 2008) and the accessibility of services (Mayston et al., 2016), together with social support such as supplementary income, can sustain community inclusion (Lamb and Goertzel, 1977), giving sustainability to Deinstitutionalisation. Adequate coordination across community-based services allowed the adequate externalization of users with complex needs (Cohen, 1983; Conway et al., 1994; Evans et al., 2012). Scaled-up outpatient facilities including local acute hospitals and intermediate facilities (Bennett and Morris, 1983; Abas et al., 2003) were key in allowing mental health systems to reduce their reliance on inpatient care and limiting beds in psychiatric settings (PAHO, 2008). Plans to end seclusion and to support mental health professionals toward a transformation in their clinical practice were identified as a facilitator to the transition (Ash et al., 2015).

Other facilitators included the continuity of care after discharge (Sytema et al., 1996) and specific actions such as: developing mobile teams and home interventions as they facilitate access to service for users who cannot physically access needed services (John et al., 2010), mitigating self-stigma dynamics by allowing an active participation of users in their treatment through shared decision-making with professional staff (Chan and Mak, 2014; Mayston et al., 2016; Matsea et al., 2019) and supporting mechanisms for primary care workers such as a 24 h hotline for assistance when it is required (Huang et al., 2017).

In terms of training, it is argued that a reform such as PDI requires the development of an educational infrastructure including local health training networks for continuing education and training needs, and targeting providers, service-users, volunteers, family members and others (Wasylenki and Goering, 1995). The incorporation of non-specialized, community-based workers trained on mental health prevention and promotion is also highlighted (Mayston et al., 2016).

Expanding user’s freedom to choose among service options was a central facilitator. This includes models of self-directed care, where users are given a budget to choose between service options (Kalisova et al., 2018). Experiences from the US, Germany and England show that patients used their budget to pay for care from their relatives, avoiding the use of institutionalized settings and preventive care options, thus shifting from crisis intervention to early interventions (Alakeson, 2010). Self-directed care improved user’s autonomy and has proved to be an effective preventive intervention (Alakeson, 2010).

#### Workforce

Facilitators related to community mental health services workforce were organized under this category. Strategies around training and skills include enhancing psychiatric aspects in health curriculum and provision of grants to complete training and research projects. This attracted students from other professions to the community mental health field (Weiss, 1990). Having previous experience in general medicine before training into psychiatry appeared to support a culture of community-based work and a strong collaboration with primary care teams (PAHO, 2008).

#### Exogenous factors

Factors indirectly affecting the feasibility of implementing Deinstitutionalisation policies were gathered under this category. This includes the role of exogenous shocks (e.g., conflict and humanitarian disasters) (Shen and Snowden, 2014) in bringing wider public attention to patients’ living conditions. A study also mentioned how the end of dictatorial regimes brought attention to human rights issues in psychiatric care, facilitating the process of Deinstitutionalisation in countries such as Argentina, Brazil and Chile (PAHO, 2008).

## Discussion

A marked decline in interest on psychiatric institutions across the global mental health literature has been noted by Cohen and Minas (2017) being absent from important prioritization exercises like the Grand Challenges in Global Mental Health (Collins et al., 2011). The authors argue that although establishing high-quality community mental health services is crucial for improving the lives of people with severe mental disorders, an exclusive focus scalability overlooks ongoing deficiencies in treatment quality and human rights protections in psychiatric institutions. Given their role in human rights abuses experienced by people with mental disorders, PDI efforts should receive more attention.

In response to this call, this article organized the available evidence around PDI, to assist in planning and conducting contextually relevant studies about and for the process. Drawing on the review, the following section introduces a set of proposals while reflecting on the limitations and problems with the available literature.

### Moving psychiatric deinstitutionalization forward

The transition from a system centered on long-term psychiatric hospital care to one centered on community-based services is complex, usually prolonged and requires adequate planning, sustained support and careful intersectoral coordination. The literature documenting and discussing PDI processes is vast, running across different time periods, regions, socio-political circumstances, and disciplines, and involving diverse models of institutional and community-based care. Based on this scoping review, we propose five key considerations for researchers and policymakers involved in PDI efforts:

1. *Needs assessment, design and scaling up.* An adequate assessment of the institutionalized population is required, to shape existing and new community-based services around their needs and preferences. A thorough analysis of the correlation of forces required to unlock institutional inertia is crucial.
2. *Financing the transition.* A comprehensive and sustainable investment is necessary, and the different aspects of the transition should be adequately costed, including new facilities, support of independent living, training, new professional roles, and the reinforcement of primary health care.
3. *Workforce development.* The workforce should be aligned with the transition from the outset. Elements such as training, incentives and guarantees of job stability are required. Curricular changes in psychiatric training, including more emphasis on community-based care and recovery-oriented practices, are necessary.
4. *PDI implementation.* The implementation process requires political resolve, careful monitoring, and an ability to respond to unexpected challenges. PDI represents a crucial learning opportunity for further scaling up.
5. *Monitoring and quality assurance.* Results of the process need to be carefully assessed against clear operational goals. The perspectives of users, caregivers, and the workforce should be incorporated into the assessments. The development of an assessment strategy detailing clear outcomes that incorporate financial and organizational dimensions is advised. Thorough documentation of PDI process, including achievements and setbacks should be done to build a reliable and diverse evidence-base for action.

A multifaceted strategy, clear and strong leadership, participation from diverse stakeholders and long-term political and financial commitment are basic elements in the planning of PDI processes. Nonetheless, implementation dynamically responds to local conditions, widely differing across countries and regions. What appears as a barrier or a facilitator can vary according to a specific context.

Although this review focuses on the barriers and facilitators for processes of PDI, we recognize that outcomes are important, and they cannot be separated from processes. Misconceptions about outcomes can hinder PDI efforts, and failed processes can lead to negative outcomes.

Two misconceptions are common. The first suggests a strong correlation between decreasing psychiatric beds and increasing homelessness or imprisonment among people with mental health problems. However, in their analysis of 23 cohort studies, Winkler et al. (2016) found that homelessness and imprisonment occurred only sporadically, and, in most studies, cases of homelessness or imprisonment were not reported.

The second misconception considers that PDI can be negative for formerly institutionalized individuals. In his review on the impact of deinstitutionalization on discharged long-stay patients, mainly diagnosed with schizophrenia, Kunitoh (2013) found that most studies reported favorable changes in social functioning, stability and improvements in psychiatric symptoms, and positive changes in quality of life and participant attitudes toward their environment, at various time-points. Deterioration following deinstitutionalization was rare. This suggests that even long-stay patients, who commonly experience functional impairment due to schizophrenia, can achieve better functioning through deinstitutionalization.

At the same time, failure at the level of process – including planning and implementation – can lead to negative and even fatal outcomes for patients. In South Africa, from October 2015 to June 2016, a poorly executed attempt to relocate 1,711 highly dependent patients resulted in 144 deaths and 44 missing individuals (Freeman, 2018). This tragedy stemmed from ethical, political, legal, administrative, and clinical errors. Reports examining this failure offer valuable lessons for PDI efforts globally (Wessels and Naidoo, 2021).

### Limitations in the literature: Time, space, process and voice

The literature on PDI is diverse, which makes synthesis endeavors difficult. Although promoted as a global standard in psychiatric and social care, the multiplicity of contexts in which the policy has been implemented limits the possibility of finding common ground. In their systematic review of the current evidence on mental health and psychosocial outcomes for individuals residing in mental health-supported accommodation services, McPherson et al. (2018) noted how the variation in service models, the lack of definitional consistency, and poor reporting practices in the literature stymie the development of adequate synthesis.

Similarly, in a recent systematic review of psychiatric hospital reform in LMICs, Raja et al. (2021, p. 1355) expressed regret over the “dearth of research on mental hospital reform processes,” indicating how poor methodological quality and the existence of variation in approach and measured outcomes challenged the extrapolation of findings on the process or outcomes of reform. Of the 12 studies they selected, 9 of them were rated as weak according to their quality assessment.

Beyond the challenges posed to synthesis efforts and through conducting this review, we identified four wider problems affecting the literature documenting PDI planning and implementation. They are related to *time*, *location*, *focus*, and *voice.*

In terms of *time*, most of the work addressing PDI was developed at the end of the 1970s through the 1980s and early 1990s. After this, there are barriers and facilitators documented which indirectly relate to the development of community-based services and their evaluation, with PDI as the “background” but not as the main object of attention. Also, the date of the search – May 2020 – could potentially exclude studies that worked with data from the pre-COVID period.

When it comes to *location*, while there is a wealth of literature on the topic, it is important to note that much of it is based on the experiences of the USA and Western Europe. The documentation of PDI in regions outside of the “global north” is typically limited to personal testimonies from process leaders, which may lack systematicity and are usually published in languages other than English. This can restrict their accessibility and dissemination.

In terms of *focus*, most studies have a clinical orientation, evaluating various outcomes that are directly or indirectly related to PDI. However, the process itself, has received little attention. An exclusive emphasis on outcomes can obscure the administrative, legal, and political complexities of carrying out a psychiatric reform, this, hinder the dissemination of important lessons.

Finally, it is worth noting that important *voices* are often missing from available studies and reports on PDI processes. While some studies do consider the experiences and engagement of caregivers, healthcare workers, and patients, they are still in the minority. This can create a skewed understanding of the impact of PDI, as these individuals play crucial roles in shaping the process and its outcomes. The same goes for the different communities where patients have developed their lives after PDI.

These limitations have significant consequences. It is unclear whether the evidence extracted from experiences in high-income countries in North America and Europe can directly inform processes in other regions, including low- and middle-income countries (LMICs). While it is possible to identify common pitfalls, barriers, and needs, this identification must be accompanied by up-to-date local research to ensure that the evidence is relevant and applicable to specific contexts.

The involvement of patients and communities affected by institutionalization in the design and implementation of research and policy should be central in a renewed PDI agenda. The recently launched Guidelines on deinstitutionalization, including in emergencies, by the United Nations Committee on the Rights of Persons with Disabilities represent a pioneering effort in this direction (OHCHR, 2022).

At the same time, qualitative and ethnographically oriented case studies are required to closely examine PDI efforts while remaining attentive to diversity and local creativity beyond global normative parameters of success and failure. Furthermore, reflexive, and flexible approaches to research synthesis are necessary to capture and assess the wealth of lessons learned from diverse engagements with deinstitutionalization across the globe.

This article offers a preliminary and general classification of barriers and facilitators that can inform the development of relevant research through various methodologies and other literature. The categories can be modified and customized based on the evidence from various settings. As far as we know, this classification is not yet present in the existing literature.

## Conclusion

Institutional models of care continue to dominate mental health service provision and financing in many countries, leading to a continued denial of the right to freedom and a life in the community for individuals with mental health conditions and associated disabilities. The successful implementation of PDI requires detailed planning, sustained support and coordinated action across different sectors.

This review identifies the factors impacting PDI processes, according to the available literature. Barriers and facilitators are organized in 15 thematic groups. The results reveal that PDI processes are complex and multifaceted, requiring detailed planning and commensurate financial and political support. We have offered five considerations for policymakers and researchers interested and/or involved in PDI efforts.

There are many lessons to be learned from the processes described in the literature, and many areas where research has been insufficient. Barriers and facilitators will differ in response to the legal, institutional, and political characteristics of each region and country. This categorization can be adapted to national realities and different levels of policy progress in PDI, to guide research and policy efforts. We call for methodological innovation and the involvement of affected communities as key elements of a renewed research agenda around this neglected aspect of mental health reform worldwide.

## Data Availability

The authors confirm that the data supporting the findings of this study are available within the article (and/or its Supplementary Materials).

## Supplementary material

For supplementary material accompanying this paper visit https://doi.org/10.1017/gmh.2023.18. click here to view supplementary material

## References

[r1] Abas M, Vanderpyl J, Le Prou T, Kydd R, Emery B and Foliaki SA (2003) Psychiatric hospitalization: Reasons for admission and alternatives to admission in South Auckland, New Zealand. Australian & New Zealand Journal of Psychiatry 37(5), 620–625. 14511092 10.1046/j.1440-1614.2003.01229.x

[r2] Adauy MH, Angulo LP, Sepúlveda AMJ, Sanhueza XA, Becerra ID and Morales JV (2013) Barreras y facilitadores de acceso a la atención de salud: Una revisión sistemática cualitativa. Revista Panamericana de Salud Publica/Pan American Journal of Public Health 33(3), 223–229. 23698142 10.1590/s1020-49892013000300009

[r3] Aggett P and Goldberg D (2005) Pervasive alienation: On seeing the invisible, meeting the inaccessible and engaging “lost to contact” clients with major mental illness. Journal of Interprofessional Care 19(2), 83–92. 15823883 10.1080/13561820400024092

[r4] Alakeson V (2010) International development in self-directed care. Issue brief, the Commonwealth Fund. Issues in International Health Policy 78, 1–11. 20232527

[r5] Allen DG, Lowe K, Moore K and Brophy S (2007) Predictors, costs and characteristics of out of area placement for people with intellectual disability and challenging behaviour. Journal of Intellectual Disability Research 51(6), 409–416. 17493024 10.1111/j.1365-2788.2006.00877.x

[r6] Anderson LL, Lakin KC, Mangan TW and Prouty RW (1998) State institutions: Thirty years of depopulation and closure. Mental Retardation 36(6), 431–443. 9879181 10.1352/0047-6765(1998)036<0431:SITYOD>2.0.CO;2

[r7] Arksey H and O’Malley L (2005) Scoping studies: Towards a methodological framework. International Journal of Social Research Methodology Theory and Practice 8(1), 19–32.

[r8] Ash D, Suetani S, Nair J and Halpin M (2015) Recovery-based services in a psychiatric intensive care unit - The consumer perspective. Australasian Psychiatry 23(5), 524–527. 26148737 10.1177/1039856215593397

[r9] Barbui C, Papola D and Saraceno B (2018) Forty years without mental hospitals in Italy. International Journal of Mental Health Systems 12(1), 1–9. 30079100 10.1186/s13033-018-0223-1 PMC6069799

[r10] Barry KL, Blow FC, Dornfeld M and Valenstein M (2002) Aging and schizophrenia: Current health services research and recommendations. Journal of Geriatric Psychiatry and Neurology 15(3), 121–127. 12230081 10.1177/089198870201500302

[r11] Barton WE (1983) The place, if any, of the mental hospital in the community mental health care system. Psychiatric Quarterly 55(2–3), 146–155. 6679601 10.1007/BF01064848

[r12] Bennett D and Morris I (1983) Deinstitutionalization in the United Kingdom. International Journal of Mental Health 11(4), 5–23.

[r13] Bredenberg K (1983) Residential integration of mentally able and elderly mentally ill patient. Psychiatric Quarterly 55(2–3), 192–205. 6679606 10.1007/BF01064853

[r14] Bryant W, Craik C and McKay EA (2004) Living in a glasshouse: Exploring occupational alienation. Canadian Journal of Occupational Therapy 71(5), 282–289. 10.1177/000841740407100507 15633878

[r15] Campbell C and Burgess R (2012) The role of communities in advancing the goals of the movement for global mental health. Transcultural Psychiatry 49(4), 379–395. 23008350 10.1177/1363461512454643

[r16] Chakraborty A, King M, Leavey G and McKenzie K (2011) Perceived racism, medication adherence, and hospital admission in African-Caribbean patients with psychosis in the United Kingdom. Social Psychiatry & Psychiatric Epidemiology 46(9), 915–923. 20607213 10.1007/s00127-010-0261-8

[r17] Chan KKS and Mak WWS (2014) The mediating role of self-stigma and unmet needs on the recovery of people with schizophrenia living in the community. Quality of Life Research 23(9), 2559–2568. 24756436 10.1007/s11136-014-0695-7

[r18] Chopra P and Herrman HE (2011) The long-term outcomes and unmet needs of a cohort of former long-stay patients in Melbourne, Australia. Community Mental Health Journal 47(5), 531–541. 20931282 10.1007/s10597-010-9351-z

[r19] Cohen GD (1983) Psychogeriatric program in a public housing setting. Psychiatric Quarterly 55(2–3), 173–181. 6679603 10.1007/BF01064850

[r20] Cohen A and Minas H (2017) Global mental health and psychiatric institutions in the 21st century. Epidemiology and Psychiatric Sciences 26(1), 4–9. 27641449 10.1017/S2045796016000652 PMC6998654

[r21] Collins PY, Patel V, Joestl SS, March D, Insel TR, Daar AS and Walport M (2011) Grand challenges in global mental health. Nature 475(7354), 27–30. 21734685 10.1038/475027a PMC3173804

[r22] Conway AS, Melzer D and Hale AS (1994) The outcome of targeting community mental health services: Evidence from the west Lambeth schizophrenia cohort. British Medical Journal (International Edition) 308(6929), 627–630. 10.1136/bmj.308.6929.627a PMC2539712 8148711

[r23] Dorwart R, Schlesinger M, Davidson H, Epstein S and Hoover C (1991) A National Study of psychiatric hospital care. American Journal of Psychiatry 148(2), 204–210. 1987819 10.1176/ajp.148.2.204

[r24] Evans A, Okeke B, Ali S, Achara-Abrahams I, Ohara T, Stevenson T, Warner N, Bolton C, Lim S, Faith J, King J, Davidson L, Poplawski P, Rothbard A and Salzer M (2012) Converting partial hospitals to community integrated recovery centers. Community Mental Health Journal 48, 557–563. 22015957 10.1007/s10597-011-9449-y

[r25] Fakhoury W and Priebe S (2002) The process of deinstitutionalisation: An international overview. Current Opinion in Psychiatry 15(2), 187–192.

[r26] Fisher K, Haagen B and Orkin F (2005) Acquiring medical services for individuals with mental retardation in community-based housing facilities. Applied Nursing Research 18(3), 155–159. 16106333 10.1016/j.apnr.2004.08.006

[r27] Freedman RI and Moran A (1984) Wanderers in a promised land. The chronically mentally ill and deinstitutionalization. Medical Care 22(12), S1–S60. 6439956

[r28] Freeman MC (2018) Global lessons for deinstitutionalisation from the ill-fated transfer of mental health-care users in Gauteng, South Africa. The Lancet Psychiatry 5(9), 765–768. 30026060 10.1016/S2215-0366(18)30211-6

[r29] Goering P, Wasylenki M, Lancee W and Freeman S (1984) From hospital to community: Six month and two-year outcomes for 505 patients. The Journal of Nervous and Mental Disease 172(11), 667–673. 6092532 10.1097/00005053-198411000-00005

[r31] Gostin LO (2008) ‘Old’ and ‘new’ institutions for persons with mental illness: Treatment, punishment or preventive confinement? Public Health 122(9), 906–913. 18555496 10.1016/j.puhe.2007.11.003

[r32] Grabowski DC, Aschbrenner KA, Feng Z and Mor V (2009) Mental illness in nursing homes: Variations across states. Health Affairs 28(3), 689–700. 19414877 10.1377/hlthaff.28.3.689 PMC2777514

[r33] Harden A (2010) Mixed-methods systematic reviews: Integrating quantitative and qualitative findings. Focus, technical brief: A publication of the National Center for the dissemination of disability research. NCDDR 25, 1–8.

[r34] Hillman AA (2005) Human rights and deinstitutionalisation: A success story in the Americas. Revista Panamericana de Salud Publica/Pan American Journal of Public Health 18(4–5), 374–379. 16354435 10.1590/s1020-49892005000900018

[r35] Huang H, Poremski D, Goh YL, Hendriks M and Fung D (2017) Increasing the continuity of care between primary care Provider and a psychiatric Hospital in Singapore. East Asian Archives of Psychiatry 27(4), 156–161. 29259146

[r36] Hudson CG (2019) Deinstitutionalisation of mental hospitals and rates of psychiatric disability: An international study. Health & Place 56, 70–79. 30710836 10.1016/j.healthplace.2019.01.006

[r37] John SM, Venkatesan S, Tharyan A and Bhattacharji S (2010) The challenge of patients with severe psychiatric illness who do not access care - A way forward. Tropical Doctor 40(4), 247–248. 20826590 10.1258/td.2010.100133

[r38] Kaffman M, Nitzan D and Elizur Y (1996) Bridging individual, family and community care: A comprehensive treatment program for the chronic mentally ill. Israel Journal of Psychiatry & Related Sciences 33(3), 144–157. 9009514

[r39] Kalisova L, Pav M, Winkler P, Michalec J and Killaspy H (2018) Quality of care in long-term care departments in mental health facilities across the Czech Republic. European Journal of Public Health 28(5), 885–890. 30084999 10.1093/eurpub/cky151

[r40] Kleiner RJ and Drews D (1992) Community-based treatment of psychiatric disorders in USA and Norway: Insights for new service delivery systems. International Journal of Social Psychiatry 38(2), 95–106. 1506142 10.1177/002076409203800203

[r41] Kormann RJ and Petronko MR (2004) Community inclusion of individuals with behavioral challenges: Who supports the care providers? Mental Retardation 42(3), 223–228. 15117224 10.1352/0047-6765(2004)42<223:CIOIWB>2.0.CO;2

[r42] Kraudy E, Liberati A, Asioli F, Saraceno B and Tognoni G (1987) Organization of services and pattern of psychiatric care in Nicaragua: Result of a survey in 1986. Acta Psychiatrica Scandinavica 76(5), 545–551. 3434326 10.1111/j.1600-0447.1987.tb02917.x

[r43] Kunitoh N (2013) From hospital to the community: The influence of deinstitutionalization on discharged long-stay psychiatric patients. Psychiatry and Clinical Neurosciences 67(6), 384–396. 23890091 10.1111/pcn.12071

[r44] Lamb HR and Goertzel V (1977) The long-term patient in the era of community treatment. Archives of General Psychiatry 34(6), 679–682. 326216 10.1001/archpsyc.1977.01770180065005

[r45] Lavoie-Tremblay M, Bonin JP, Bonneville-Roussy A, Briand C, Perreault M, Piat M, Lesage A, Racine H, Laroche D and Cyr G (2012) Families’ and decision makers’ experiences with mental health care reform: The challenge of collaboration. Archives of Psychiatric Nursing 26(4), 41–50. 10.1016/j.apnu.2012.04.007 22835756

[r46] Lean M, Fornells-Ambrojo M, Milton A, Lloyd-Evans B, Harrison-Stewart B, Yesufu-Udechuku A, Kendall T and Johnson S (2019) Self-management interventions for people with severe mental illness: Systematic review and meta-analysis. British Journal of Psychiatry 214(5), 260–268. 10.1192/bjp.2019.54 PMC6499726 30898177

[r47] Lucas PJ, Baird J, Arai L, Law C and Roberts HM (2007) Worked examples of alternative methods for the synthesis of qualitative and quantitative research in systematic reviews. BMC Medical Research Methodology 7, 1–7. 17224044 10.1186/1471-2288-7-4 PMC1783856

[r48] Mallik K, Reeves RJ and Dellario DJ (1998) Barriers to community integration for people with severe and persistent psychiatric disabilities. Psychiatric Rehabilitation Journal 22(2), 175–180.

[r49] Manuel JI, Hinterland K, Conover S and Herman DB (2012) “I Hope I can make it out there”: Perceptions of women with severe mental illness on the transition from hospital to community. Community Mental Health Journal 48(3), 302–308. 21997644 10.1007/s10597-011-9442-5

[r50] Matsea T, Ryke E and Weyers M (2019) Stakeholders’ views regarding their role as support system for people with mental illness and their families in rural South Africa. Community Mental Health Journal 55(4), 672–679. 30238282 10.1007/s10597-018-0337-6

[r51] May P, Lombard Vance R, Murphy E, O’Donovan MA, Webb N, Sheaf G, McCallion P, Stancliffe R, Normand C, Smith V and McCarron M (2019) Effect of deinstitutionalisation for adults with intellectual disabilities on costs: A systematic review. BMJ Open 9(9), 1–9. 10.1136/bmjopen-2018-025736 PMC6756329 31542732

[r52] Mays N, Roberts E and Popay J (2001) Synthesising research evidence. In Fulop N, Allen P, Clarke A and Black N (eds.), Studying the Organisation and Delivery of Health Services: Research Methods. London: Routledge, pp. 188–220.

[r53] Mayston R, Alem A, Habtamu A, Shibre T, Fekadu A and Hanlon C (2016) Participatory planning of a primary care service for people with severe mental disorders in rural Ethiopia. Health Policy and Planning 31(3), 367–376. 26282860 10.1093/heapol/czv072 PMC5007595

[r54] McCubbin M (1994) The illusion of disillusion. The Journal of Mind and Behavior 15(1/2), 35–53.

[r55] McPherson P, Krotofil J and Killaspy H (2018) Mental health supported accommodation services: A systematic review of mental health and psychosocial outcomes. BMC Psychiatry 18(1), 1–15. 29764420 10.1186/s12888-018-1725-8 PMC5952646

[r56] Mechanic D and Rochefort D (1990) Deinstitutionalization: An appraisal of reform. Annual Review of Sociology 16, 301–327.

[r57] Mezzina R, Rosen A, Amering M and Javed A (2019) The practice of freedom: Human rights and the global mental health agenda. In Advances in Psychiatry. Cham: Springer, pp. 483–515.

[r58] Moseneke D (2018) In the Arbitration Between: Families of Mental Health Care Users Affected by the Gauteng Mental Marathon Project, and National Minister of Health of the Republic of South Africa. Johannesburg: Government of the Province of Gauteng, Premier of the Province of Gauteng.

[r59] O’Doherty S, Linehan C, Tatlow-Golden M, Craig S, Kerr M, Lynch C and Staines A (2016) Perspectives of family members of people with an intellectual disability to a major reconfiguration of living arrangements for people with intellectual disability in Ireland. Journal of Intellectual Disabilities 20(2), 137–151. 26968194 10.1177/1744629516636538

[r60] OHCHR (2022) Guidelines on Deinstitutionalization, including in Emergencies (Conventions on the Rights of Persons with Disabilities). United Nations. Available at https://tbinternet.ohchr.org/_layouts/15/treatybodyexternal/Download.aspx?symbolno=CRPD/C/5. (accessed January 2023).

[r61] Oshima I and Kuno E (2006) Living arrangements of individuals with schizophrenia in Japan: Impact of community-based mental health services. International Journal of Social Psychiatry 52(1), 55, 40–64. 10.1177/0020764006061249 16463595

[r62] PAHO (2008) Innovative Mental Health Programs in Latin America & The Caribbean (ed. Caldas J and Cohen A) Washington, DC: PAHO.

[r65] Peters M, Godfrey C, Khalil H, McInerney P, Parker D and Soares C (2015) Guidance for conducting systematic scoping reviews. International Journal of Evidence-Based Healthcare 13(3), 141–146. 26134548 10.1097/XEB.0000000000000050

[r66] Raja T, Tuomainen H, Madan J, Mistry D, Jain S, Easwaran K and Singh SP (2021) Psychiatric hospital reform in low-and middle-income countries: A systematic review of literature. Social Psychiatry and Psychiatric Epidemiology 56, 1341–1357. 33884439 10.1007/s00127-021-02075-z PMC8316186

[r67] Richardson A, Richard L, Gunter K and Derrett S (2019) Interventions to integrate care for people with serious mental illness and substance use disorders: A systematic scoping review protocol. BMJ Open 9(10), 1–7. 10.1136/bmjopen-2019-031122 PMC6830830 31666268

[r68] Rizzardo R, Rovea A and Magni G (1986) The general practitioner and the psychiatric health service in Italy after the reform: Opinions and experiences in an urban district. Acta Psychiatrica Scandinavica 73(3), 234–238. 3716840 10.1111/j.1600-0447.1986.tb02679.x

[r69] Rose S (1979) Deciphering deinstitutionalization: Complexities in policy and program analysis. The Milbank Memorial Fund Quarterly 57(4), 429–460. 114875

[r70] Rosenheck R (2000) The delivery of mental health services in the 21st century: Bringing the community back in. Community Mental Health Journal 36(1), 107–124. 10708049 10.1023/a:1001860812441

[r71] Sade R, Sashidharan S and Silva M (2021) Paths and detours in the trajectory of the Brazilian psychiatric reform. Salud Colectiva 17, e3563. 35896314 10.18294/sc.2021.3563

[r72] Saraceno B (2003) La liberación de los Pacientes psiquiátricos de la rehabilitación Psicosocial a la ciudadanía Posible. México: Editorial Pax.

[r73] Saraceno B, van Ommeren M, Batniji R, Cohen A, Gureje O, Mahoney J, Sridhar D and Underhill C (2007) Barriers to improvement of mental health services in low-income and middle-income countries. Lancet 370(9593), 1164–1174. 17804061 10.1016/S0140-6736(07)61263-X

[r74] Schmidt G (2000) Barriers to recovery in a first nations community. Canadian Journal of Community Mental Health 19(2), 75–87. 11381739 10.7870/cjcmh-2000-0016

[r75] Semke J (1999) Shifts in case mix and locus of mental health care for Washington state adults with severe mental illness. Administration and Policy in Mental Health 26(3), 191–205. 10339834 10.1023/a:1021362630116

[r76] Shen G and Snowden L (2014) Institutionalisation of deinstitutionalisation: A cross-national analysis of mental health system reform. International Journal of Mental Health Systems 8(1), 47. 25473417 10.1186/1752-4458-8-47 PMC4253997

[r77] Stelovich S (1979) From the hospital to the prison: A step forward in deinstitutionalization? Hospital and Community Psychiatry 30(9), 618–620. 468138 10.1176/ps.30.9.618

[r78] Swidler RN and Tauriello JV (1995) New York state community mental health reinvestment act. Psychiatric Services 46(5), 496–500. 7627677 10.1176/ps.46.5.496

[r79] Sytema S, Micciolo R and Tansella M (1996) Service utilization by schizophrenic patients in Groningen and South-Verona: An event-history analysis. Psychological Medicine 26(1), 109–119. 8643750 10.1017/s0033291700033754

[r80] Taylor Salisbury T, Killaspy H and King M (2016) An international comparison of the deinstitutionalisation of mental health care: Development and findings of the mental health services deinstitutionalisation measure (MENDit). BMC Psychiatry 16 (1), 1–10. 26926473 10.1186/s12888-016-0762-4 PMC4772656

[r81] Thomas J and Harden A (2008) Methods for the thematic synthesis of qualitative research in systematic reviews. BMC Medical Research Methodology 8, 1–10. 18616818 10.1186/1471-2288-8-45 PMC2478656

[r82] Thomas J, Harden A, Oliver S, Sutcliffe K, Rees R, Brunton G and Kavanagh J (2004) Integrating qualitative research with trials in systematic reviews. BMJ 328, 1010–1012. 15105329 10.1136/bmj.328.7446.1010 PMC404509

[r83] Thornicroft G, Deb T and Henderson C (2016) Community mental health care worldwide: Current status and further developments. World Psychiatry 15(3), 276–286. 27717265 10.1002/wps.20349 PMC5032514

[r84] Tricco A, Lillie E, Zarin W, O’Brien K, Colquhoun H, Levac D, Moher D, Peters M, Horsley T, Weeks L, Hempel S, Akl E, Chang C, McGowan J, Stewart L, Hartling L, Aldcroft A, Wilson M, Garritty C and Straus S (2018) PRISMA extension for scoping reviews (PRISMA-ScR): Checklist and explanation. Annals of Internal Medicine 169(7), 467–473. 30178033 10.7326/M18-0850

[r85] Turner T (2004) The history of deinstitutionalisation and reinstitutionalisation. Psychiatry 3(9), 1–4.

[r86] Wasylenki DA and Goering PN (1995) The role of research in systems reform. Canadian Journal of Psychiatry 40(5), 247–251. 7553543

[r87] Weiss J (1990) Ideas and inducements in mental health policy. Journal of Policy Analysis and Management 9(2), 178–200. 10104414

[r88] Wessels J and Naidoo T (2021) The management of a policy implementation project: The disastrous Gauteng mental health Marathon project. In Wessels J, Potgieter T and Naidoo T (eds), Public Administration Challenges: Cases from Africa. Cape Town: Juta, pp. 25–58.

[r89] Westman J, Gissler M and Wahlbeck K (2012) Successful deinstitutionalization of mental health care: Increased life expectancy among people with mental disorders in Finland. The European Journal of Public Health 22(4), 604–606. 21659387 10.1093/eurpub/ckr068

[r90] WHO (2013) Mental Health Action Plan 2013–2020. WHO Document Production Services. Geneva: World Health Organization.

[r91] WHO (2021a) Comprehensive Mental Health Action Plan 2013–2030. World Health Organization, Vol. 2021. Geneva: World Health Organization.

[r92] WHO (2021b) Guidance on Community Mental Health Services: Promoting Person-Centred and Rights-Based Approaches, Vol. 2021. Geneva: World Health Organization.

[r93] WHO and the Gulbenkian Global Mental Health Platform (2014) Innovation in Deinstitutionalization: A WHO Expert Survey. Geneva: World Health Organization.

[r94] Winkler P, Barrett B, McCrone P, Csémy L, Janousková M and Höschl C (2016) Deinstitutionalised patients, homelessness and imprisonment: Systematic review. The British Journal of Psychiatry 208(5), 421–428. 27143007 10.1192/bjp.bp.114.161943

[r95] Yip KS (2006) Community mental health in the People’s Republic of China: A critical analysis. Community Mental Health Journal 42(1), 41–51. 16570115 10.1007/s10597-005-9003-x

[r96] Yohanna D (2013) Deinstitutionalisation of people with mental illness: Causes and consequences. American Medical Association Journal of Ethics 15(10), 886–891. 24152782 10.1001/virtualmentor.2013.15.10.mhst1-1310

