# Moving psychiatric deinstitutionalization forward: A scoping review of barriers and facilitators – CORRIGENDUM

**DOI:** 10.1017/gmh.2023.87

**Published:** 2023-12-13

**Authors:** Cristian Montenegro, Matías Irarrázaval Dominguez, Josefa González Moller, Felicity Thomas, Jorge Urrutia Ortiz

The authors apologise that upon publication of Montenegro C, et al (2023), the funding acknowledgement was omitted. This should have been included and is listed below

Acknowledgments: This work was made possible by the financial support of the Chilean Agency for Research and Development (ANID), under the Initiation Fondecyt grant Number 11191019.

The article has been updated to include this statement.
